# Shaping the nonlinear near field

**DOI:** 10.1038/ncomms10361

**Published:** 2016-01-14

**Authors:** Daniela Wolf, Thorsten Schumacher, Markus Lippitz

**Affiliations:** 1 Experimental Physics III, University of Bayreuth, Universitätsstrasse 30, D-95440 Bayreuth, Germany; 2 Max Planck Institute for Solid State Research, Heisenbergstrasse 1, D-70569 Stuttgart, Germany

## Abstract

Light scattering at plasmonic nanoparticles and their assemblies has led to a wealth of applications in metamaterials and nano-optics. Although shaping of fields around nanostructures is widely studied, the influence of the field inside the nanostructures is often overlooked. The linear field distribution inside the structure taken to the third power causes third-harmonic generation, a nonlinear optical response of matter. Here we demonstrate by a far field Fourier imaging method how this simple fact can be used to shape complex fields around a single particle alone. We employ this scheme to switch the third-harmonic emission from a single point source to two spatially separated but coherent sources, as in Young's double-slit assembly. We envision applications as diverse as coherently feeding antenna arrays and optical spectroscopy of spatially extended electronic states.

## Abstract (web summary)

While shaping of the electromagnetic fields around nanostructures is widely studied, the influence of the field inside the nanostructures is often overlooked. Here, Wolf *et al*. control the spatial distribution of third-harmonic emission in a plasmonic nanostructure, imaged by a far field Fourier method.

## Graphical abstract

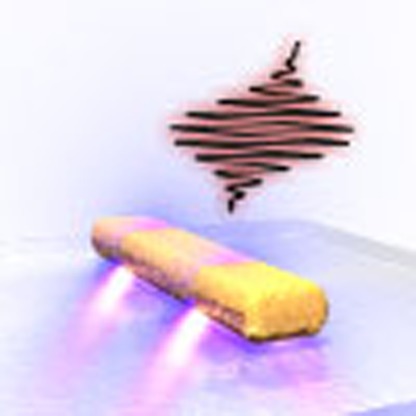

When a noble metal nanostructure is excited by ultrafast laser pulses, nonlinear optical effects such as higher harmonics generation or multiphoton-induced luminescence can be observed^1^,^2^,^3^,^4^. The correlation between linear and nonlinear response^5^,^6^, the spectral dependence of the nonlinear signal^7^,^8^ and the use of nanoantennas to boost optical nonlinearities^9^,^10^ has been investigated in detail. Although the linear response of plasmonic nanostructures is well understood and almost any desired field distribution can be realized by well-chosen arrangements of multiple particles^11^,^12^,^13^, the spatial origin of nonlinear signals such as second-harmonic^14^,^15^ and third-harmonic generation (THG)^16^,^17^,^18^ is still under debate. Spatial features that are below the diffraction limit of the fundamental wavelength can still be resolved at the shorter wavelength of the higher harmonic signals^15^,^19^. However, real-space imaging does not give access to the coherence properties of different emitting spots. In Young's double-slit experiment^20^, the coherent emission from two slits interferes due to the wave nature of light and a characteristic pattern of dark and bright stripes can be observed in the far field. The emerging Fraunhofer diffraction pattern corresponds to the Fourier transform of the apertures^21^. The shape and intensity distribution of the interference pattern in the far field is thus uniquely related to both width and separation of the slits, as well as their relative phase.

We set out to control the spatial distribution of third-harmonic emission in a plasmonic nanostructure. A schematic overview of the experiment is given in the introductory Fig. 1. In analogy to Young's experiment, the emission properties manifest in the far field interference pattern, allowing a reconstruction of the nonlinear near fields. Using this method, we prove that it is the field inside the structure and not the external hotspots that cause THG. As illustrated in Fig. 1, we show that the emission of a gold rod can be switched between a configuration with one single and two separated sources when the excitation wavelength is tuned over a higher-order plasmon resonance. Our results are supported by calculations of the emission patterns using single dipoles, as well as simulations of the linear and nonlinear fields using a finite element method.

## Results

### Nonlinear plasmonic analogue of a single slit

**Figure 2:**
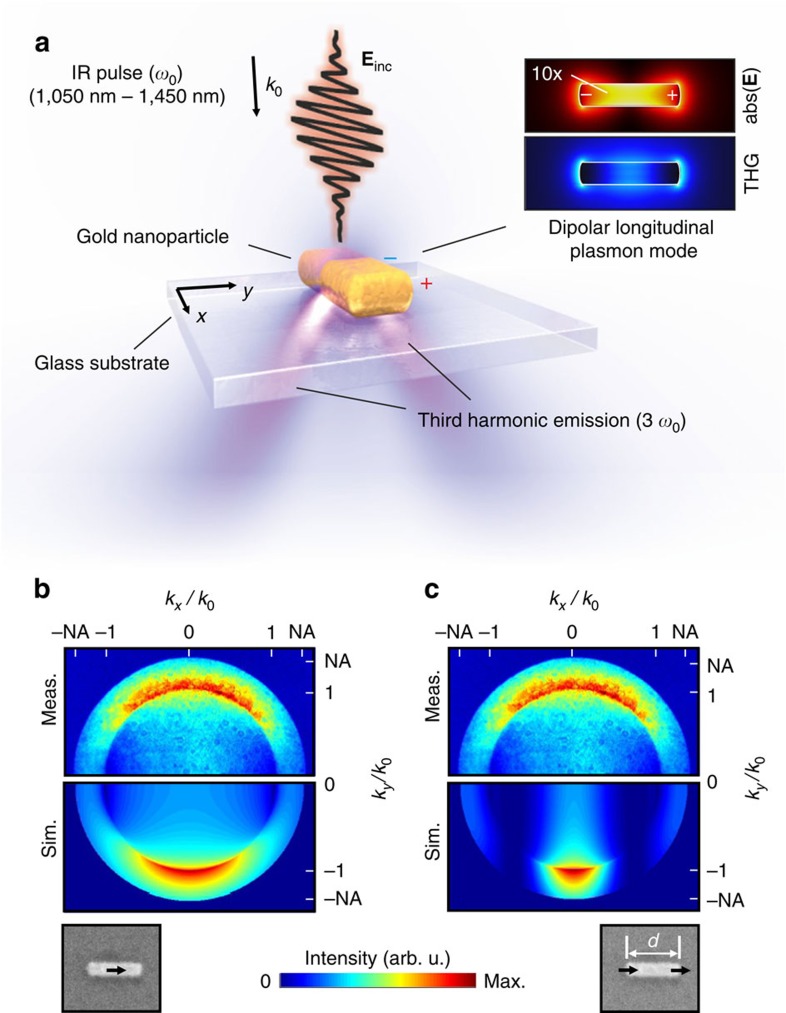
Localizing the emission from a single nanorod. (**a**) Schematic representation of the measurement method. A single gold nanoparticle is excited by infrared light from the air side and the generated third harmonic is detected through the glass substrate. Inset: electric field and resulting third-harmonic field of a nanorod excited at the fundamental plasmon resonance calculated with a finite element method. The linear field inside the particle is scaled up by a factor of 10 for better visibility. (**b**,**c**) Measured back focal plane image of a 270-nm-long rod compared with two hypotheses: (**b**) the emission stems from one spot in the centre, modelled as one dipole or (**c**) the emission stems from the end surfaces, modelled by two equal, in phase dipoles, separated by *d*=270 nm. Shown is always one half of the symmetric emission pattern. Excitation wavelength is 1,170 nm.

To introduce our experimental technique, we first discuss the nonlinear plasmonic analogue of a single slit as illustrated in Fig. 2a. A single 270-nm-long gold nanorod on a glass substrate is illuminated by a focused beam of short near-infrared laser pulses, leading to THG. Other nonlinear signals are filtered spectrally (see Supplementary Fig. 1). At 1,170 nm wavelength, the fundamental dipolar plasmon mode is excited resonantly in this rod, as can be seen in Fig. 2a from the field distribution calculated with a finite element method. Regarding the THG, the field inside the particle is the crucial parameter^17^. The internal field is highest right in the centre of the rod, which is in stark contrast to the field outside the particle, peaking at the ends of the rod. As the nonlinear material polarization is proportional to the third power of this internal field^1^, the calculation predicts that the third-harmonic signal is emitted from the centre of the structure for this geometry. In free space, the angular radiation pattern would be that of a free dipole, that is, of toroidal shape with the nanorod being on the symmetry axis. The air–glass interface refracts and reflects part of this emission so that a typical two-lobed angular pattern remains in the direction of the glass substrate^22^. We detect this angular emission pattern by imaging the back focal plane of the microscope objective that collects the light emitted into the substrate (see Methods section). The field distribution in the back focal plane is the Fourier transform of the sample plane^21^, corresponding thus to the Fraunhofer pattern in the classical diffraction experiments. The finite acceptance angle *α* of the microscope objective given by the numerical aperture NA=*n* sin (*α*) limits the observable region in reciprocal space to |*k*/*k*_0_|<NA, with *n* being the refractive index of the substrate and *k*_0_=2*π*/*λ* the wave vector in free space. As shown in Fig. 2b, we find good agreement between the measured emission pattern of the nanorod and the calculated pattern of a single dipole oriented parallel to the interface, corresponding to emission from the centre of the particle. Assuming two dipoles oscillating in phase as in Fig. 2c, corresponding to emission from the ends of the rod, clearly yields a different radiation pattern (for full emission patterns, see Supplementary Fig. 2 and for quantitative analysis see Supplementary Fig. 3). We therefore conclude that it is the high field inside the gold structure that causes THG. The emission properties of a single resonantly excited nanorod can simply be described by a single dipole oriented parallel to an interface (see Supplementary Fig. 4).

### Nonlinear plasmonic analogue of Young's double slit

**Figure 3:**
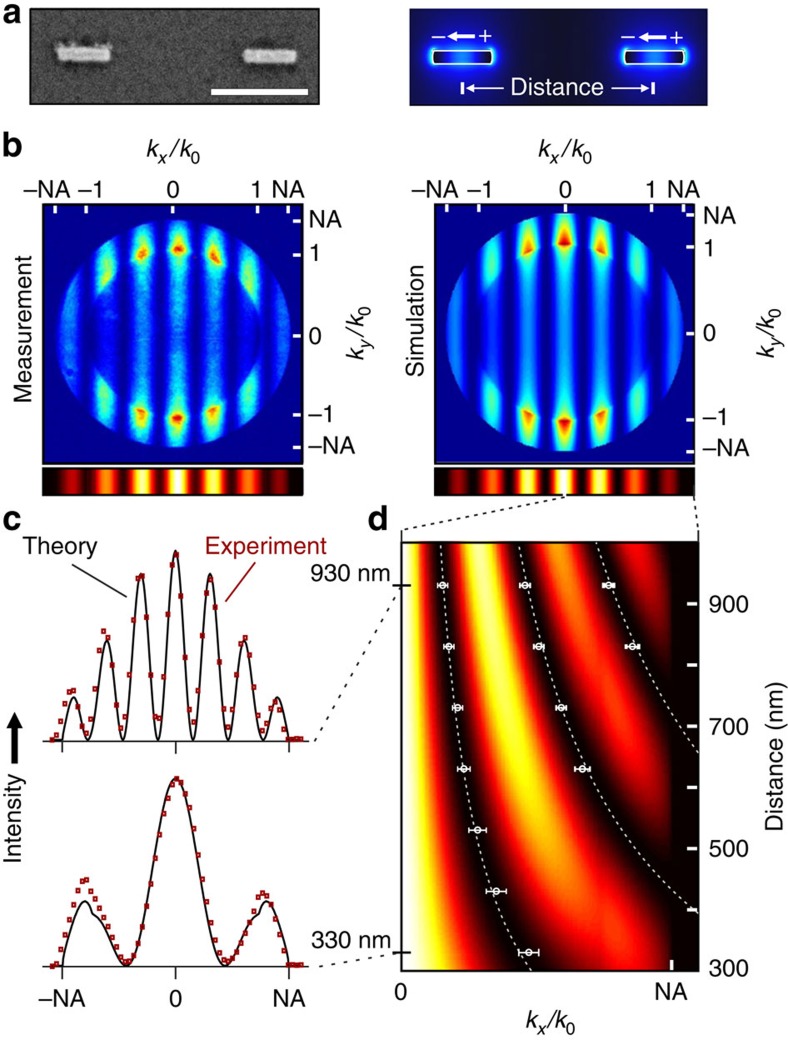
Interference in the emission of two rods. (**a**) Scanning electron microscopic image of a pair of 270-nm-long nanorods; scale bar, 500 nm. The centre distance is varied between 330 and 930 nm. According to the finite element method calculations, the particles act as two separated dipoles oscillating in phase. (**b**) Measured and calculated radiation patterns for 930 nm distance with intensity projection onto the *k*_*x*_ axis. (**c**) Measured (red squares) and calculated (black lines) intensity profiles for 330 and 930 nm distance, corresponding to cuts through the distance-dependent intensity projection shown in **d**. The squares and dashed lines indicate the positions of the minima from measurement and calculation, respectively. The error bars correspond to an increase to three times the noise level above the respective minimum.

We now turn to the nonlinear plasmonic analogue of Young's double-slit experiment. In the classical experiment, a double-slit assembly is illuminated by spatially coherent light. In our plasmonic analogue, we exploit the coherence of the third-harmonic emission from two spatially separated nanorods excited by the same laser focus (Fig. 3a). Each nanorod emits similar to a single dipole, as confirmed by numerical simulations. For a pair of identical rods, an interference pattern is superimposed on the dipolar radiation pattern of the individual rods. As shown in Fig. 3b for a distance of 930 nm, again we find good agreement between the patterns from experiment and dipole model, regarding both the position of the extrema and the intensity distribution as well. To further analyse the data, we project the back focal plane images onto the *k*_*x*_ axis, which retains all relevant details on the interference process (for the full data set, see Supplementary Fig. 5). The resulting intensity profiles for two different rod distances are shown in Fig. 3c, corresponding to the typical intensity patterns in the classical experiments. The number and especially the exact position of the minima in Fig. 3d depend strongly on the separation of the emitting centres but agree well between measurements and simulations for rod pairs with distances between 330 and 930 nm. It is noteworthy that the gap between the two particles is always larger than 60 nm so that plasmonic coupling can be excluded. The careful analysis of the interference pattern thus allows us to accurately measure the emitter distance. That way, this experiment further confirms the centres of the rods as sources of the nonlinear signal (see also Supplementary Figs 2 and 3).

### Nonlinear emission properties of a long rod

**Figure 4:**
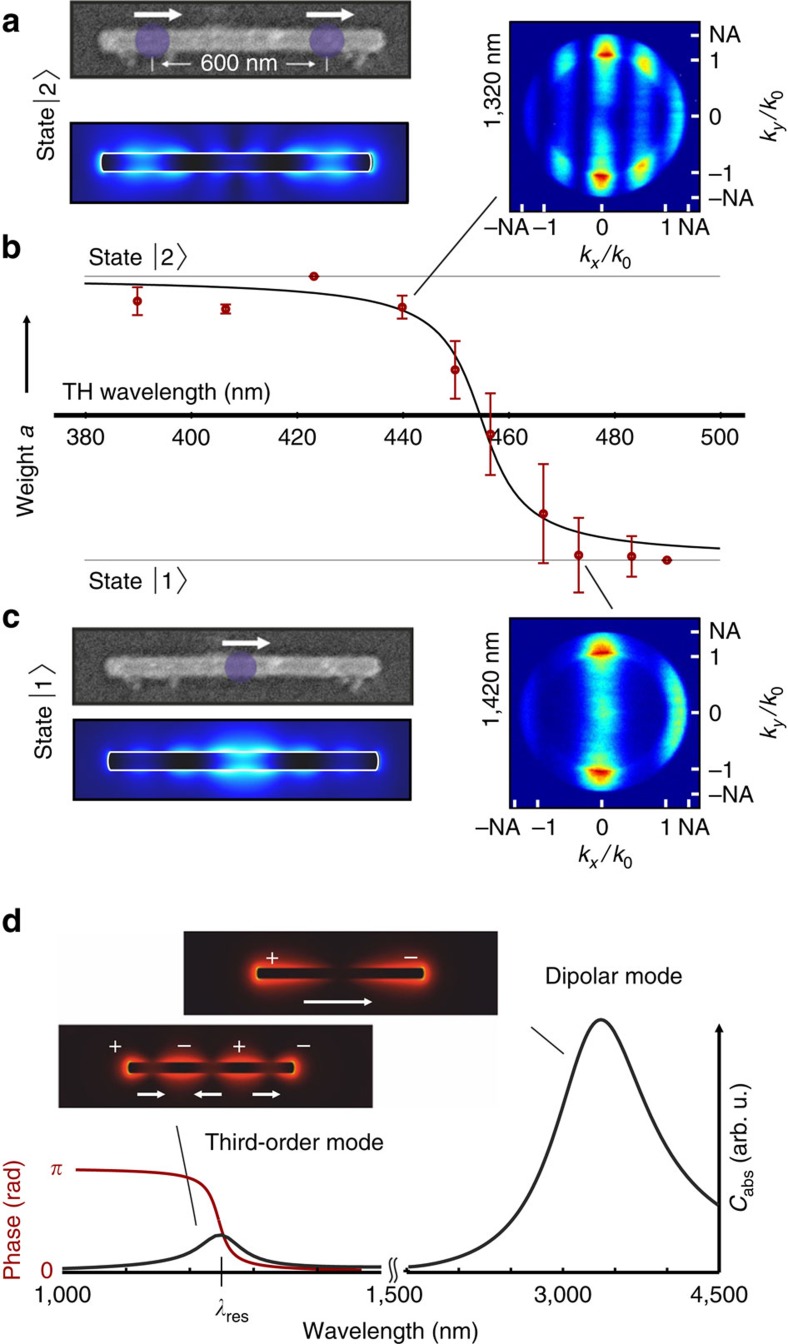
Switching of nonlinear emission and near fields. (**a**,**c**) The emission pattern of a 925-nm-long rod depends on the excitation wavelength. The two states 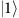 and 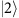 differ in the number of emitting spots, as indicated in the scanning electron microscopic images and the calculated TH fields. (**b**) We observe a transition within a wavelength range of about 25 nm for the weight *a* of state 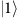 (red dots). The error bars are three times the s.d. of the fit. The black line is a guide to the eye. (**d**) Calculated absorption cross-section (black line) and phase of the third-order mode (red line) of a 925-nm-long rod. Insets: linear field distributions at the fundamental and third-order resonance.

This fact becomes even more apparent when instead of two short rods a single long rod is investigated. We demonstrate that the nonlinear emission and near field can be switched between one and two emitting centres by slightly tuning the fundamental wavelength. The experiment is summarized in Fig. 4. The angular emission pattern of a 925-nm-long gold rod at an excitation wavelength of 1,320 nm clearly displays the characteristic interference pattern of a double slit where both apertures emit in phase (see Fig. 4a). With the method presented above, the separation of the emitting centres can be determined to approximately 600 nm, as indicated in the scanning electron microscopic image. When the fundamental wavelength is tuned to 1,420 nm, we obtain an angular pattern that deviates only slightly from a single dipole, implying a dominant emission from the centre of the rod (see Fig. 4c). Our conclusions are confirmed by numerical simulations of equivalent rod structures as shown in Fig. 4a,c.

We take the emission patterns at 490 and 425 nm TH wavelength as templates for the states 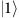 and 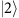, respectively, and fit the patterns at all other wavelengths by a linear superposition 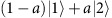 (see Supplementary Note 1 for explanation of the fitting method). As shown in Fig. 4b, the weight *a* obtained in this way displays a steep transition within 25 nm at the third-harmonic wavelength between a single emitting spot in the centre (state 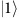) and two in-phase spots with well-defined separation (state 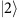).

To explain the switching of the emission pattern, we need to consider in more detail the modes of the fundamental field. The modes of long nanorods resemble standing waves, where only odd modes can be excited optically in our configuration^23^. Here, the dipolar mode is shifted far into the infrared, while the third-order mode shows a resonance in the wavelength regime where the experiments are carried out (see Fig. 4d). When the excitation wavelength is tuned over a plasmon resonance, the phase changes by *π*. In the vicinity of the third-order resonance, the phase of the third-order mode undergoes this change, whereas the phase of the dipolar mode is unaffected. Tuning the fundamental wavelength over the third-order resonance thus changes the relative phase of the two modes by *π*. It is noteworthy that the third-order resonance of the investigated structure is red-shifted compared with the calculation due to fabrication inaccuracies. The field distribution of the dipolar and the third-order mode can be described by a single dipole and by three counter-oscillating dipoles, respectively. We always excite both modes with varying efficiency and observe their superposition. For wavelengths below the resonance, the overall phase between the modes vanishes. In the dipole picture, the single dipole and the inner dipole of the third-order mode oscillate against each other and cancel. As only the outer dipoles remain, this corresponds to the double slit behaviour. Above the resonance, the overall phase between the modes is *π*. Hence, the single dipole and the inner dipole of the third-order mode add up so that the centre dipole dominates. The nonlinear third-order process of THG amplifies amplitude and phase differences of the fundamental field inside the rod and thus drastically changes the third-harmonic emission properties (see Supplementary Fig. 6). Evidently, the generated near field switches accordingly as demonstrated by the numerical simulations shown in Fig. 4, whereas only slight changes are observed in the linear near field. The same switching behaviour is observed when tuning the plasmon resonance via the length of the nanorod and keeping the excitation wavelength fixed (see also Supplementary Fig. 6).

## Discussion

In summary, we have localized spatially the third-harmonic emission centres of a single plasmonic nanorod by analysing interference in the far field. The experiment unambiguously shows that it is not the high field around the tips of the nanorod, but the standing waves inside the rod that cause the emission at the third-harmonic wavelength. This allows us to control and tune the optical near field patterns at the third harmonic solely by slight variations of the incoming field. Although optical near fields at the fundamental wavelength are affected as well, the optical nonlinearity drastically amplifies the effect. We demonstrated a switching of the emission centres by a wavelength shift of only 25 nm.

Our experiments open up a new direction for nanophotonics^24^. The local field around plasmonic nanostructures can not only be sculptured by well-chosen arrangements of many nanoparticles, but with a similar efficiency also by an engineered distribution of the fundamental field inside a continuous piece of metal. Owing to the high optical nonlinearities of noble metals^16^, a conceivable field strength at the third harmonic is obtained, resulting in unprecedented control over the placement of light sources on the nanoscale. We envision applications as diverse as coherently feeding antenna arrays^25^ or optical circuits^26^,^27^ and optical spectroscopy of spatially extended electronic states^28^.

## Methods

### Experimental set-up and sample fabrication

The signal output of a Ti:Sapphire pumped optical parametric oscillator (76 MHz, 150 fs, 1,050–1,450 nm) is focused onto the sample with an infrared lens (NA 0.55), leading to a spot size of ∼1.5 μm on the sample. The gold nanostructures are fabricated on 170-μm-thick glass coverslips using electron beam lithography followed by metal evaporation and lift-off. All structures are 60 nm wide and 30 nm high. Their length is 270 nm for the short rods and 925 nm for the long rod. The polarization of the excitation is chosen parallel to the long axis of the nanorods. Excitation powers are 2.5 and 10 mW for the short and long rods, respectively.

The transmitted light and the generated higher harmonic is collected by an oil-immersion objective (NA 1.35). The near-infrared excitation light is eliminated with a Schott KG5 filter. A back focal plane image is acquired on the charge-coupled device camera by placing an additional lens (focal length 150 mm), acting as a Bertrand lens, in front of the spectrograph. For the measurements at 1,170 nm excitation wavelength, a narrow bandpass filter transmits only the third harmonic at 390 nm and the back focal plane images are acquired using the spectrograph with a mirror instead of a grating. For the wavelength-dependent measurements, the bandpass filter is removed and a grating disperses the light. The convolution of spatial pattern and emission spectrum has little influence, as the emission spectrum is very peaked. To locate the structures, the sample is scanned with a piezo stage and the emitted light is detected by an avalanche photodiode. In this case, the Bertrand lens is removed.

### Simulation methods

To calculate the radiation patterns, we assume a dipole in air (*n*=1) at a height of 15 nm above the glass interface (*n*=1.5). We calculate the fields from the Fresnel coefficients and project the resulting intensity distribution onto the back focal plane^22^,^29^. When two to four dipoles are considered, all dipoles emit with the same amplitude and phase. The fields from the individual dipoles are superimposed to obtain the total field, which is again projected onto the back focal plane.

For the finite element calculations, we use the commercial software package ‘Comsol Multiphysics'. The simulations for the linear (at *ω*_0_) and nonlinear (at 3*ω*_0_) response are performed in frequency domain and separated into two models. In both, the dimensions of the structures are identical and matched to those in the experiment. As dielectric function of gold, we use the data reported by Johnson and Christy^30^. For the linear response, we assume a plane wave excitation and solve Maxwell's equations for the given boundary value problem. The shown linear field distributions are the absolute values of the electric field **E**(*ω*_0_) in a plane 15 nm above the substrate (*n*=1.5). The third-harmonic polarization is calculated as described in previous work^17^ from 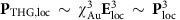. To obtain the third-harmonic fields around the structure, we use the third-harmonic polarization as surface boundary value at the gold structures and solve the system at 3*ω*_0_. The third-harmonic field plots show the absolute values of the nonlinear electric field **E**(3*ω*_0_) in the same plane as the linear fields before.

In Supplementary Table 1, we provide information about which simulation method has been used in the individual graphs in this paper.

## Additional information

**How to cite this article:** Wolf, D. *et al*. Shaping the nonlinear near field. *Nat. Commun.* 7:10361 doi: 10.1038/ncomms10361 (2016).

## Supplementary Material

Supplementary Information Supplementary Figures 1–6, Supplementary Table 1 and Supplementary Note 1.

## Figures and Tables

**Figure 1:**
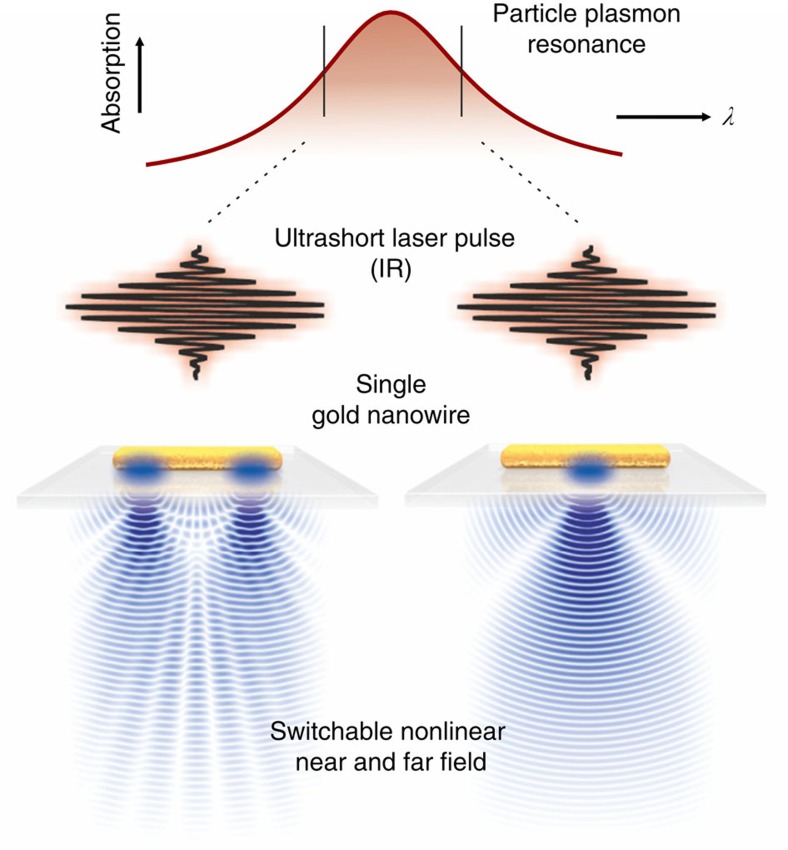
Nonlinear near field and emission control. Slightly tuning the excitation wavelength over a plasmon resonance drastically changes the local fields and thus the far field response of a simple plasmonic structure. In our experiment, we switch between a configuration with one single and two separated emitting centres. In analogy to classical diffraction experiments, the coherent emission from different sources leads to characteristic interference patterns in the far field. We exploit this effect to determine the position and relative phase of emitting centres in a single plasmonic nanoparticle and the concomitant nonlinear near fields.

## References

[b1] Boyd R. W. Nonlinear Optics 3rd edn Academic Press (2008).

[b2] Lippitz M., Dijk M. A. & Orrit M. Third-harmonic generation from single gold nanoparticles. Nano Lett. 5, 799–802 (2005). 15826131 10.1021/nl0502571

[b3] Renger J., Quidant R., Van Hulst N. & Novotny L. Surface-enhanced nonlinear four-wave mixing. Phys. Rev. Lett. 104, 046803 (2010). 20366728 10.1103/PhysRevLett.104.046803

[b4] Kauranen M. & Zayats A. V. Nonlinear plasmonics. Nat. Photonics 6, 737–748 (2012).

[b5] Hentschel M., Utikal T., Giessen H. & Lippitz M. Quantitative modeling of the third harmonic emission spectrum of plasmonic nanoantennas. Nano Lett. 12, 3778–3782 (2012). 22686215 10.1021/nl301686x

[b6] O'Brien K. . Predicting nonlinear properties of metamaterials from the linear response. Nat. Mater. 14, 379–383 (2015). 25664451 10.1038/nmat4214

[b7] Hanke T. . Efficient nonlinear light emission of single gold optical antennas driven by few-cycle near-infrared pulses. Phys. Rev. Lett. 103, 257404 (2009). 20366283 10.1103/PhysRevLett.103.257404

[b8] Metzger B., Hentschel M., Lippitz M. & Giessen H. Third-harmonic spectroscopy and modeling of the nonlinear response of plasmonic nanoantennas. Opt. Lett. 37, 4741–4743 (2012). 23164898 10.1364/ol.37.004741

[b9] Harutyunyan H., Volpe G., Quidant R. & Novotny L. Enhancing the nonlinear optical response using multifrequency gold-nanowire antennas. Phys. Rev. Lett. 108, 217403 (2012). 23003302 10.1103/PhysRevLett.108.217403

[b10] Aouani H., Rahmani M., Navarro-Ca M. & Maier S. A. Third-harmonic-upconversion enhancement from a single semiconductor nanoparticle coupled to a plasmonic antenna. Nat. Nanotechnol. 9, 1–5 (2014). 24608232 10.1038/nnano.2014.27

[b11] Prodan E., Radloff C., Halas N. J. & Nordlander P. A hybridization model for the plasmon resonance of complex nanostructures. Science 302, 419–422 (2003). 14564001 10.1126/science.1089171

[b12] Schuller J. A. . Plasmonics for extreme light concentration and manipulation. Nat. Mater. 9, 193–204 (2010). 20168343 10.1038/nmat2630

[b13] Luk'yanchuk B. . The Fano resonance in plasmonic nanostructures and metamaterials. Nat. Mater. 9, 707–715 (2010). 20733610 10.1038/nmat2810

[b14] Valev V. K. . Asymmetrical optical second-harmonic generation from chiral G-shaped gold nanostructures. Phys. Rev. Lett. 104, 127401 (2010). 20366565 10.1103/PhysRevLett.104.127401

[b15] Mascheck M. . Observing the localization of light in space and time by ultrafast second-harmonic microscopy. Nat. Photonics 6, 293–298 (2012).

[b16] Utikal T. . Towards the origin of the nonlinear response in hybrid plasmonic systems. Phys. Rev. Lett. 106, 133901 (2011). 21517383 10.1103/PhysRevLett.106.133901

[b17] Metzger B., Schumacher T., Hentschel M., Lippitz M. & Giessen H. Third harmonic mechanism in complex plasmonic Fano structures. ACS Photonics 1, 471–476 (2014). 25540812 10.1021/ph5000677 PMC4270418

[b18] Liu X., Larouche S., Bowen P. & Smith D. R. Clarifying the origin of third-harmonic generation from film-coupled nanostripes. Opt. Express 23, 19565–19574 (2015). 26367614 10.1364/OE.23.019565

[b19] Hanke T. . Tailoring spatiotemporal light confinement in single plasmonic nanoantennas. Nano Lett. 12, 992–996 (2012). 22268812 10.1021/nl2041047

[b20] Young T. A Course of Lectures on Natural Philosophy and the Mechanical Arts vol. 1, (Printed for J. Johnson (1807).

[b21] Goodman J. W. Introduction to Fourier Optics 3rd edn MaGraw-Hill (2005).

[b22] Novotny L. & Hecht B. Principles of Nano-Optics 2nd edn Cambridge University Press (2012).

[b23] Dorfmüller J. . Plasmonic nanowire antennas: experiment, simulation, and theory. Nano Lett. 10, 3506–3603 (2010). 20726567 10.1021/nl101921y

[b24] Koenderink A. F., Alù A. & Polman A. Nanophotonics: Shrinking light-based technology. Science 348, 516–521 (2015). 25931548 10.1126/science.1261243

[b25] Dregely D. . Imaging and steering an optical wireless nanoantenna link. Nat. Commun. 5, 4354 (2014). 24993946 10.1038/ncomms5354 PMC4102110

[b26] Engheta N. Circuits with light at nanoscales: optical nanocircuits inspired by metamaterials. Science 317, 1698–1702 (2007). 17885123 10.1126/science.1133268

[b27] Gramotnev D. K. & Bozhevolnyi S. I. Plasmonics beyond the diffraction limit. Nat. Photonics 4, 83–91 (2010).

[b28] Dubin F. . Macroscopic coherence of a single exciton state in a polydiacetylene organic quantum wire. Nat. Phys. 2, 32–35 (2006).

[b29] Lieb M. A., Zavislan J. M. & Novotny L. Single-molecule orientations determined by direct emission pattern imaging. J. Opt. Soc. Am. B 21, 1210–1215 (2004).

[b30] Johnson P. B. & Christy R. W. Optical constants of noble metals. Phys. Rev. B 6, 4370–4379 (1972).

