# Supplementary material for: Shaping the nonlinear near field
Source: Nat Commun. 2016 Jan 14;7:10361. doi: 10.1038/ncomms10361 (PMC4735599; doi:10.1038/ncomms10361)
Supplement: Supplementary Information — Supplementary Figures 1–6, Supplementary Table 1 and Supplementary Note 1. [file ncomms10361-s1.pdf]

Supplementary figures

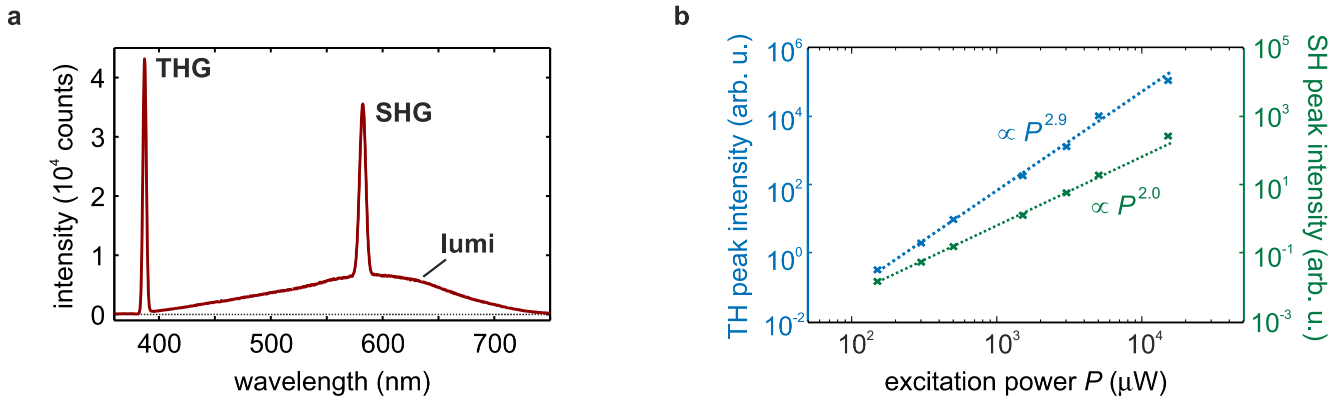

SUPPLEMENTARY FIG. 1: **Spectrum and power dependence.** a) Spectrum of a single 270 nm long gold nanorod, excited at 1170 nm with approx. 3 mW. The third and second harmonic peaks can be identified at 390 nm and 580 nm, respectively. Additionally, there is a broad luminescence background between 400 and 750 nm. As second harmonic generation is forbidden in centrosymmetric materials like gold and structures with inversion symmetry, we attribute the SH signal from our sample to imperfections such as defects and surface roughness. With the SHG process being more efficient than the THG process, this leads to a comparably large signal at the second harmonic. However, the amplitudes of the SH signal vary strongly from particle to particle. b) Power dependence of the signal at the third (blue) and second (green) harmonic. Plotted is the peak intensity of the signals at each power.

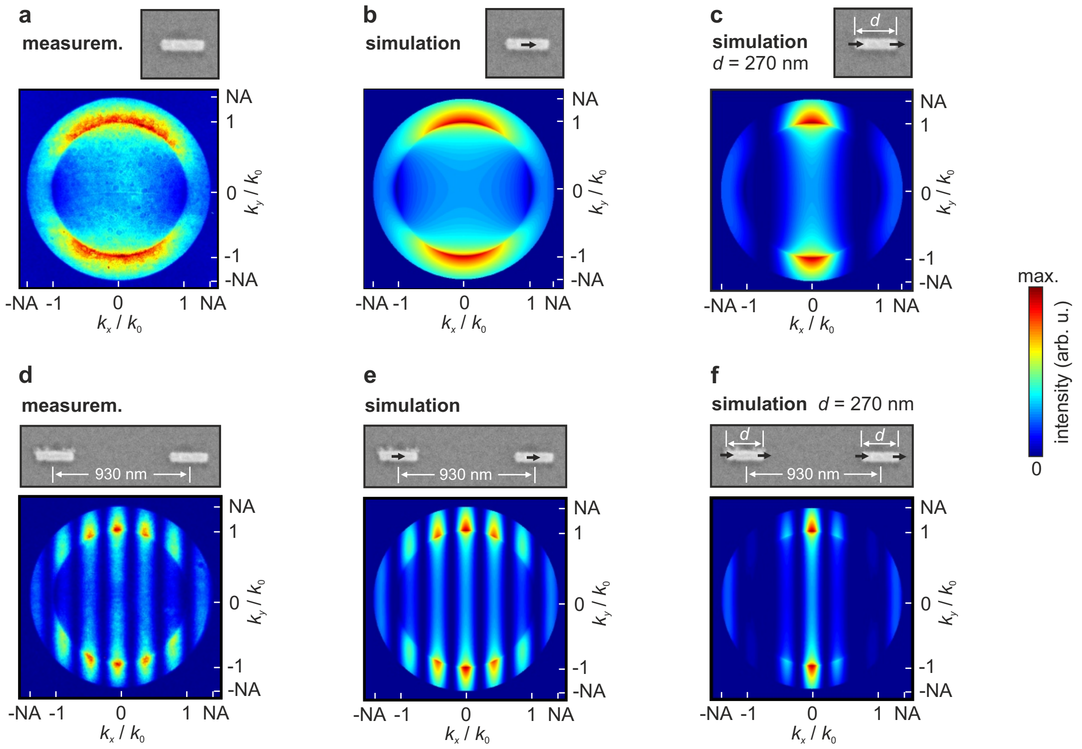

SUPPLEMENTARY FIG. 2: **Emission patterns of the single slit and double slit analogue.** The full emission patterns of the measurement and the simulations with one and with two dipoles are shown for the single 270 nm long rod in (a)-(c) and the double rod structure in (d)-(f). The measured data shows good agreement with the calculations with one dipole in the centre of each rod. This excludes emission from the ends of the structures.

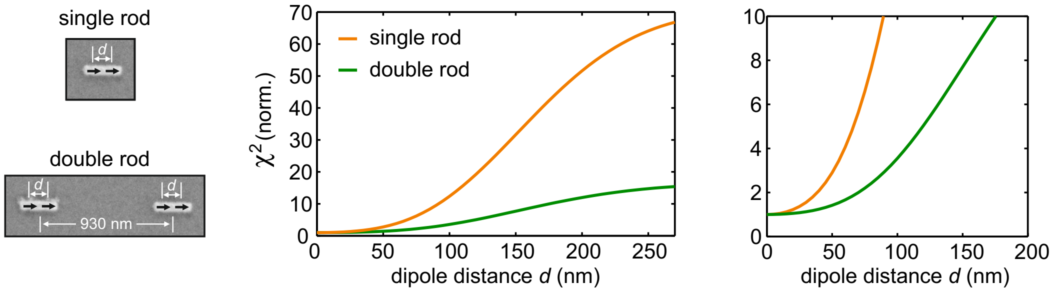

SUPPLEMENTARY FIG. 3: **Deviation between measured and calculated intensity profiles.** To further prove that we can distinguish between emission from the center of the rods and emission from the ends, the χ^2 deviation of the calculated intensity profile from the measured profile is plotted for the single and the double rod structure. Each rod is represented by two dipoles with varying distance between 0 nm (corresponding to emission from the center) and 270 nm (corresponding to emission from the ends). The χ^2 deviation is given by $\chi^2 = \sum (I_c - I_m)^2$ where I_c are the data points of the calculated profile and I_m the data points from the measurement. Both intensity profiles (I_c, I_m) are normalised to the same integral value before comparing data and simulation. The plotted χ^2 is normalized such that $\chi^2 = 1$ at $d = 0$ nm. Especially the close-up view on the right shows strong deviations for dipole distances even below 100 nm, which excludes emission from the ends of the rods.

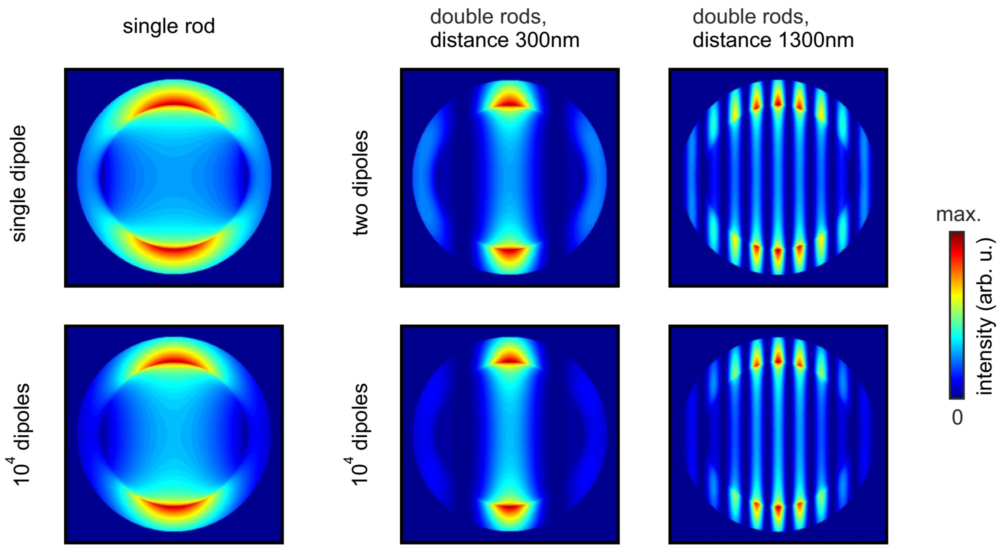

SUPPLEMENTARY FIG. 4: **Comparison of different number of dipoles.** Top row: calculated emission patterns from a single dipole corresponding to a single rod structure and two dipoles corresponding to a double rod structure. Bottom row: calculated emission patterns from an array of many dipoles (1 nm grid) where the amplitude and phase of the dipoles corresponds to the nonlinear polarization calculated with the finite element method. The difference between the patterns obtained with the different methods is marginal, supporting our assumption of single point-like dipoles.

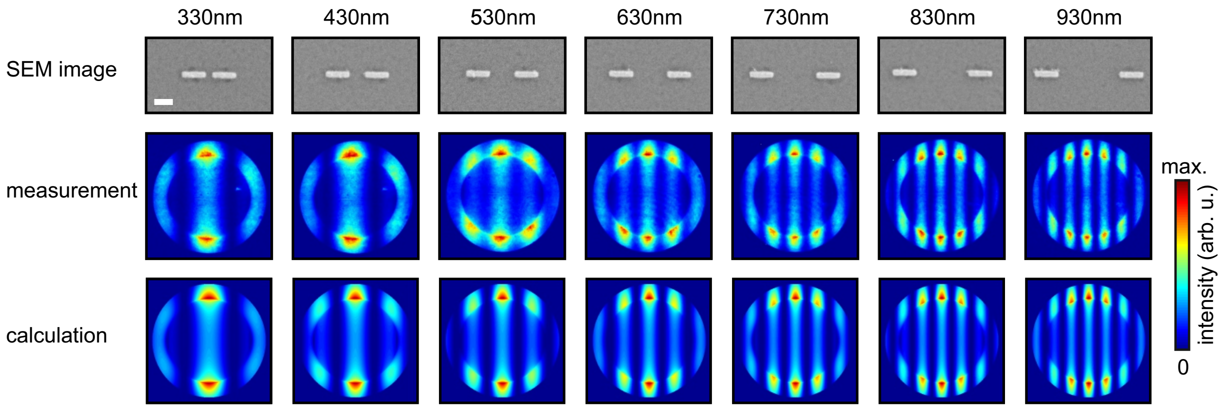

SUPPLEMENTARY FIG. 5: **Distance dependence of the plasmonic double-slit.** The full series of the measured and calculated radiation patterns of our nonlinear plasmonic double-slit analogue. The centre distance of two 270 nm long gold rods is varied between 330 and 930 nm, as shown in the SEM images. The middle row shows the measured radiation patterns, the bottom row the calculated patterns for two dipoles with the same separation. With increasing distance, the number of side maxima and minima increases. Scale bar is 200 nm.

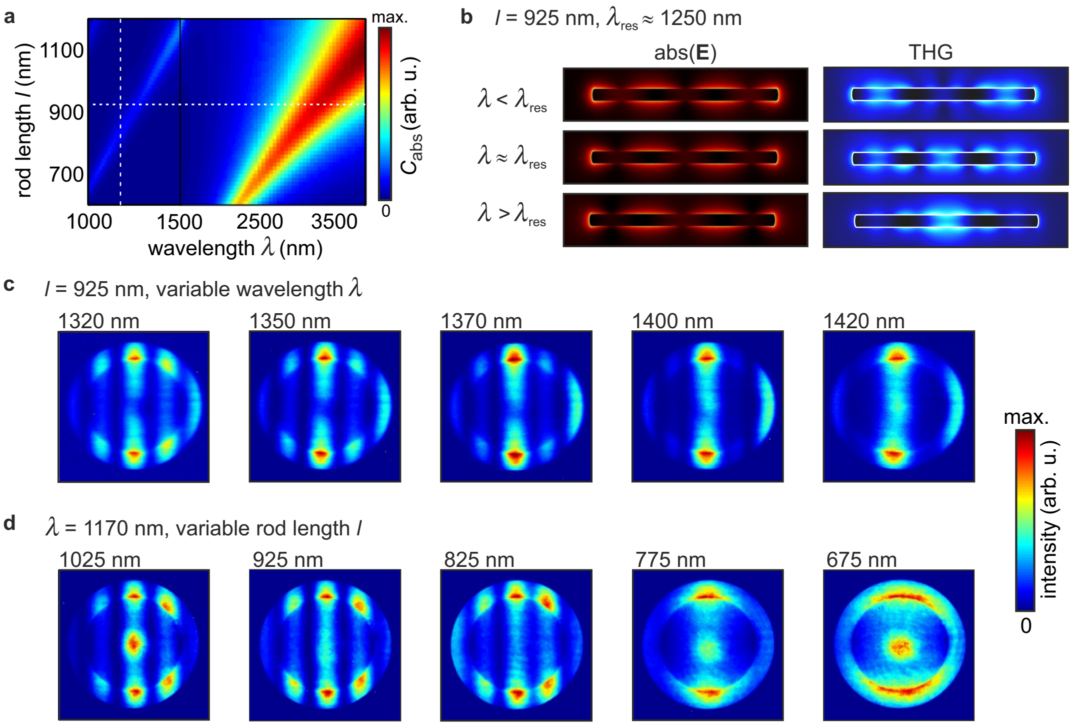

SUPPLEMENTARY FIG. 6: **Switching in extended rod structures.** a) Calculated absorption cross section for different nanorod lengths. The wavelength regime has been divided into two parts with different resolution at 1500 nm, indicated by the solid black line. The dipolar plasmon resonance is at 2.5 - 3.5 μm while the third-order resonance appears in the wavelength regime considered in our experiments. The third-order plasmon resonance can be crossed in two ways as indicated by the dashed white lines; either by changing the excitation wavelength for a fixed rod length or by changing the rod length for a fixed excitation wavelength. b) Calculated linear and third-harmonic fields of a 925 nm long rod structure in the vicinity of the third-order plasmon resonance. While the linear fields are almost unaffected, the amplification by the third power leads to a switching from a double-slit to a single-slit configuration at the third harmonic. On resonance, a superposition of both configurations is observed. c) Radiation patterns measured at different wavelengths for a fixed rod length of 925 nm and d) at a fixed excitation wavelength of 1170 nm for different rod lengths. In both cases, the transition from a double-slit pattern to a dipole pattern is observed, as predicted by the simulation. Outside the wavelength region shown in c, no qualitative change in the radiation pattern is observed. This also justifies the choice of the base functions for the fitting routine described in the Supplementary Note. The red-shift of the third-order resonance in the experiment compared to the calculation is due to fabrication inaccuracies.

Supplementary Table 1: Assignment of the simulation methods

	element description	method used
Fig. 2a	field plots	finite elements
Fig. 2b	emission patterns	single point dipoles (1)
Fig. 2c	emission patterns	single point dipoles (2)
Fig. 3a	field plots	finite elements
Fig. 3b-d	emission patterns, intensity projection and profiles	single point dipoles (2)
Fig. 4a,c	field plots	finite elements
Fig. 4b	field plots, absorption spectrum	finite elements
Fig. S 2	emission patterns	single point dipoles (1, 2, 4)
Fig. S 3	deviation curve of intensity profile	single point dipoles (2, 4)
Fig. S 4	emission patterns	single point dipoles (1, 2, 10^4)
Fig. S 5	emission patterns	single point dipoles (2)
Fig. S 6a	absorption cross section	finite elements
Fig. S 6b	field plots	finite elements

SUPLLEMENTARY TABLE 1: **Assignment of the used calculation methods to the individual graphs.** The numbers in the brackets indicate the number of point dipoles used. When an array of many hundred dipoles is used, the relative amplitudes and phases are chosen according to the nonlinear polarization calculated with the finite element method. In all other cases, amplitude and phase of all dipoles are the same.

Supplementary Note 1: Fitting of the switching behaviour

We describe the switching behaviour in the emission characteristics of a 925 nm long nanorod as a linear superposition $(1-a)\,|1\rangle + a\,|2\rangle$ of the two limiting states. Here, $n = 1, 2$ gives the number of emitting centres in the corresponding state $|n\rangle$ and a is the weight $(0 \leq a \leq 1)$. We use the emission patterns measured at 490 nm and 425 nm as base functions for the states $|1\rangle$ and $|2\rangle$. The radiation patterns are projected onto the k_x axis and the weight a at all measured wavelengths is determined by fitting the linear superposition to the measured data using a nonlinear Levenberg-Marquardt algorithm.

The uncertainty in the fit parameter a is estimated by the algorithm and given by the square root of the diagonal elements of the covariance matrix. Three times this standard deviation is given as error bars in the plot. As the patterns at 490 nm and 425 nm were chosen as base functions, the uncertainty vanishes at these wavelengths.
